# Post-pandemic assessment of parental perceptions toward COVID-19 vaccination and general immunization—an insight from polio endemic country

**DOI:** 10.3389/fpubh.2025.1627965

**Published:** 2025-12-29

**Authors:** Muhammad Abdullah, Agha Syed Zain Haider, Ahmad Umais Ahad, Muhammad Shoaib Alam, Shaeem Tahir, Hurais Malik, Talha Bin Yasin, Muhammad Hudaib, Syed Shoaib Bukhari, Sher Afgan Ali Khan Burki, Muneeba Mushtaq, Ayat Ul Karam, Jawaria Shahzad, Rana Sajawal Joiya, Khabab Abbasher Hussien Mohamed Ahmed

**Affiliations:** 1 CMH Lahore Medical College and IOD, Lahore, Pakistan; 2 PIMS Hospital, Islamabad, Pakistan; 3 Fazaia Ruth Pfau Medical College, Karachi, Pakistan; 4 Azra Naheed Medical College, Lahore, Pakistan; 5 Faculty of Medicine, University of Khartoum, Khartoum, Sudan

**Keywords:** vaccine hesitancy, parental attitudes, COVID-19 immunization, public health perceptions, Health Belief Model, Pakistan

## Abstract

**Background:**

The COVID-19 pandemic exposed and intensified existing challenges in immunization uptake, particularly in countries like Pakistan where vaccine hesitancy persists due to historical mistrust and misinformation. This study aims to assess parental perceptions toward COVID-19 vaccination and general immunization in a post-pandemic context, using the Health Belief Model (HBM) to contextualize behavioral drivers and barriers.

**Methods:**

A cross-sectional survey was conducted from March to June 2023 at two tertiary hospitals in Pakistan—Combined Military Hospital, Lahore, and Fazaia Ruth Pfau Medical College, Karachi. Using convenience sampling, 298 parents of children aged 5–15 years completed a validated, pilot-tested questionnaire assessing demographic variables, COVID-19 vaccine perceptions, and general immunization attitudes. Data were analyzed using SPSS v26.0, employing descriptive statistics, non-parametric tests, and Spearman's correlation.

**Results:**

Of the 298 participants (64.1% male; mean age = 34.2 years), 93.6% had at least a high school education. The COVID-19 Perception Scale showed a moderate overall score (Mean = 41.3, SD = 10.1), with moderate perceived vulnerability (Mean = 13.6, S.D = 4.5), high trust in vaccine information (Mean = 17.8, SD = 6.0), but low awareness (Mean = 2.4, SD = 1.2) and willingness to vaccinate children (Mean = 3.9, SD = 1.6). The Immunization Perception Scale indicated generally positive attitudes (Mean = 1.6, SD = 0.7), though moderate hesitancy persisted (Mean = 1.7, SD = 1.2). Significant associations emerged between personal or familial COVID-19 experience and both COVID-19 perception (*r* = 0.269, *p* < 0.01) and immunization perception (*r* = 0.121, *p* < 0.05). Mapping findings to the HBM revealed gaps in cues to action and self-efficacy, despite relatively high perceived benefits.

**Conclusion:**

Parental trust in vaccine information is evident, yet awareness and pediatric vaccine uptake remain low. These findings call for context-specific, HBM-informed public health strategies that strengthen cues to action, reduce perceived barriers, and foster community trust to improve immunization outcomes in Pakistan.

## 1 Introduction

The COVID-19 pandemic has had profound global impacts, disrupting economies, healthcare systems, and social structures worldwide. The causative agent, Severe Acute Respiratory Syndrome Coronavirus 2 (SARS-CoV-2), spreads mainly through close contact, leading the World Health Organization (WHO) to declare a global public health emergency (1). By July 29, 2022, the number of reported cases had surged to 568,373,127, prompting governments to implement stringent measures such as social distancing, quarantine, and lockdowns to control the virus's spread (2). This unprecedented crisis triggered a massive international scientific effort to understand the virus's genetics and develop effective vaccines and treatments. Healthcare systems worldwide were overwhelmed, facing severe shortages of medical supplies and personnel, exacerbating the crisis (3). Economically, the pandemic caused severe disruptions in global supply chains, unprecedented unemployment rates, and significant GDP contractions across many countries. The International Monetary Fund (IMF) projected the worst recession since the Great Depression (4). Socially, lockdowns and social distancing measures led to a shift toward remote work and education, fundamentally altering daily routines and increasing reliance on digital technologies (5).

Vaccination emerged as a key strategy for controlling COVID-19, crucial for establishing herd immunity to prevent virus transmission (6). However, Pakistan, already grappling with Immunization compliance, faced unique challenges in managing the dual burden of an ongoing pandemic while addressing existing health issues such as limited healthcare infrastructure, economic instability, and social inequalities. The country's healthcare system struggled with inadequate resources, hindering effective pandemic response and vaccine distribution (7). The government, with international partners like the WHO, implemented a Strategic Preparedness and Response Plan to mitigate the virus's spread and manage its impacts, with effective vaccination as a cornerstone (8). However, Pakistan has traditionally had low vaccination rates for diseases like HBV and polio, and this trend continued with the COVID-19 immunization efforts. This deep-rooted resistance to immunization stems from historical mistrust and cultural beliefs, requiring extensive community engagement to overcome (9). Misinformation and conspiracy theories about the COVID-19 vaccine's safety and efficacy further contributed to negative perceptions in Pakistan. Historical mistrust in vaccination programs, influenced by past polio vaccination controversies, along with religious and cultural beliefs, fueled skepticism toward new vaccines (10).

Parental attitudes toward vaccination are often shaped by a complex interplay of perceived risk, perceived benefits, and perceived barriers—the very constructs described in the Health Belief Model (HBM) (11). This model provides a valuable lens through which to understand individual health behaviors, particularly vaccination. According to HBM, individuals are more likely to engage in a health behavior (such as vaccinating their children) if they believe they are susceptible to the disease (perceived susceptibility), believe the disease is severe (perceived severity), believe the benefits of the action outweigh the costs (perceived benefits), and face minimal obstacles (perceived barriers). Additionally, cues to action (such as public health messaging) and self-efficacy also play a role (12).

In a country where polio eradication efforts have been ongoing for decades, vaccine hesitancy remains a persistent issue influenced not just by cultural, social, and political factors. Thus, understanding the persistence of these misconceptions in a post-pandemic context is crucial. While the immediate threat of COVID-19 may have diminished, the attitudes formed during the pandemic could have long-lasting effects on vaccination behaviors. This study focuses on parents, who play a pivotal role in deciding whether to vaccinate their children. Assessing their current perceptions toward COVID-19 vaccination and general immunization practices will provide valuable insights into the factors influencing vaccine acceptance and compliance.

The primary objective of this study is to assess the current perceptions and attitudes of parents toward COVID-19 vaccination in Pakistan, now that the pandemic threat has receded. Additionally, by anchoring the study in the HBM, we aim to uncover the underlying belief systems that influence vaccine acceptance and resistance. The findings of this study have significant implications for public health policy and vaccination strategies in Pakistan and other polio-endemic regions. Addressing persistent misconceptions and hesitancy is essential for enhancing vaccine uptake and ensuring the success of immunization programs. By understanding the factors influencing parental perceptions, policymakers and public health officials can develop targeted interventions to build trust and improve public health outcomes.

## 2 Methodology

### 2.1 Study design and participants

This cross-sectional study commenced at three centers following ethical approval from the Ethical Review Board of Combined Military Hospital (CMH) Lahore and Fazaia Ruth Pfau Medical College (FRPMC) Hospitals, Karachi. Data collection spanned from December 2024 to March 2025. The study aimed to assess parental perceptions toward COVID-19 vaccination and general immunization post-pandemic. A convenience sampling method was employed, enabling the inclusion of readily accessible participants. The study population consisted of parents of children aged 5–15 years who were visiting the concerned hospitals for various health needs. This approach provided a diverse and comprehensive sample, reflecting different socioeconomic backgrounds and educational levels. Inclusion criteria encompassed parents who provided informed consent and were willing to participate voluntarily. This inclusivity was designed to capture a wide spectrum of perceptions, from those with high awareness of vaccination to those with potential misconceptions or hesitancy. Exclusion criteria included non-parental guardians, parents of children with medical exemptions to vaccination, and individuals who did not consent to participate. These exclusions were necessary to maintain the focus on typical parental perceptions regarding childhood immunization.

The sample size was determined using the WHO sample size calculator 2.0, aiming for a 95% confidence interval and a 5% margin of error. Based on a population proportion of 25.9% of parents who were not vaccinated, as reported by the World Health Organization (WHO) (13), the minimum sample size required was calculated to be 298 participants. All participants completed the standardized questionnaire, resulting in a 100% cooperation rate.

To minimize biases, participants were recruited from diverse settings, reducing selection bias. Anonymity of the survey and assurances of confidentiality were employed to mitigate response bias, encouraging honest and uninfluenced responses. The questionnaire was pilot-tested to identify and rectify any ambiguous or leading questions, ensuring clarity and validity. Quality assurance was further enhanced through the training of data collectors and meticulous data entry verification. Expert reviews of the survey ensured content validity, and the study was designed to provide an accurate and comprehensive understanding of parental perceptions toward COVID-19 vaccination and general immunization in a post-pandemic context. This rigorous methodology aimed to identify key factors influencing parental attitudes and inform public health strategies to improve vaccination uptake.

### 2.2 Questionnaire development and validation

The development and validation of the questionnaire for assessing the parental perception toward COVID-19 vaccination and immunization in general for their children involved a systematic and rigorous process. This process ensured the face validity, content validity, construct validity, and reliability of the instrument.

#### 2.2.1 Development of questionnaire items

The initial pool of items in the COVID-19 Vaccination and General Immunization questionnaires employed in this study was adapted and developed based on a thorough literature review, keeping in consideration a widely used theoretical framework of HBM (11, 12) for understanding health-related behaviors, particularly vaccination uptake. The COVID-19 questionnaire, initially consisting of 15 items, and the immunization questionnaire, comprising 5 items, were adapted from two validated parent studies (6, 14) after obtaining the necessary approvals. These parent studies were chosen due to their robust methodologies and relevance to our research objectives.

The COVID-19 questionnaire was designed to assess parental perceptions toward COVID-19 vaccination, while the immunization questionnaire aimed to evaluate general parental attitudes and perceptions toward routine immunization. Both questionnaires were translated into the National Language, Urdu, and provided in both English and Urdu to eliminate potential miscommunication and ensure accurate responses. To ensure content validity, experts reviewed the questionnaires for relevance, clarity, and comprehensiveness. Their feedback was instrumental in ensuring that the questions adequately covered the intended constructs and were suitable for the target population.

#### 2.2.2 Pilot testing and refinement

To ensure face validity, a pilot test was conducted by administering the questionnaires to a small sample of 20 parents representative of the target population. This pilot test aimed to identify potential issues such as confusing or ambiguous questions, response options, or formatting problems. Feedback from pilot test participants was collected and analyzed. Based on this feedback, several changes were made to the questionnaires: ambiguous questions were rephrased for clarity, and formatting adjustments were made to enhance readability. These revisions ensured that the final questionnaires were clear, comprehensive, and user-friendly.

#### 2.2.3 Validation through exploratory factor analysis (EFA)

An Exploratory Factor Analysis (EFA) was conducted to ensure the construct validity of the questionnaires. The EFA aimed to determine the underlying structure of the questionnaires and ensure their relevance and clarity for our target population. The decision to run an EFA was driven by the need to validate the factor structure of the adapted questionnaires within our specific context and to uncover any latent constructs that may not have been identified in the parent studies.

A sample of 200 participants completed the full set of survey items. Principal Component Analysis (PCA) was employed for factor extraction, identifying underlying factors that explain the common variance among the survey items. To enhance the interpretability of the factors, orthogonal rotation (Varimax) was applied to ensure that factors are uncorrelated.

For the COVID-19 questionnaire, the Kaiser–Meyer–Olkin (KMO) measure of sampling adequacy was 0.873, indicating strong sampling adequacy. Bartlett's test of sphericity was significant (*p* < 0.001), confirming the suitability of the data for factor analysis. The initial 15 items loaded on four different subscales, resulting in the reduction of one question due to low factor loading, leaving 14 items categorized into four distinct subscales. For the Immunization questionnaire, the KMO value was 0.668, indicating moderate sampling adequacy. Bartlett's test of sphericity was significant (*p* < 0.001), confirming the suitability of the data for factor analysis. The five items loaded on two subscales, with all items retained (See Supplementary Table 1).

Items with low factor loadings (< 0.30) or cross-loadings on multiple factors were removed. Additionally, items with low communality values (< 0.30) were excluded, as these indicated that the item did not share sufficient variance with other items in the factor structure. This refinement process ensured that the final questionnaires were psychometrically sound and adequately captured the constructs of interest. This meticulous process of adaptation, pilot testing, and validation ensured that the final questionnaires were reliable and valid tools for assessing post-pandemic parental perceptions toward COVID-19 vaccination and general immunization in a polio-endemic country.

#### 2.2.4 Reliability assessment

The reliability of the final questionnaire was assessed through internal consistency. Cronbach's alpha coefficients were calculated for each subscale and the overall scales, demonstrating acceptable to high-reliability levels (Cronbach's alpha > 0.7). The final instrument, validated and refined, provided a comprehensive and reliable measure of medical students' knowledge and attitudes toward geriatric medicine, suitable for use in further research and educational assessments.

Subsequently, the revised questionnaire was administered to an additional 98 participants, resulting in a final sample size of 298 participants, inclusive of the initial 200. This expanded sample size further validates the findings and ensures the robustness of the identified factors.

### 2.3 Measures

The study utilized comprehensive questionnaires to gather data on parental perceptions (refer to Supplementary Table 2). The baseline characteristics of participants were meticulously documented, including variables such as gender, age, relationship with the child/children, educational background, economic status, number of family members, number of children, cohabitation with grandparents, presence of co-morbidities in family members and children, and family history of COVID-19 diagnosis, isolation, and positive testing.

The COVID-19 Perception Scale, designed to assess parental perceptions toward COVID-19 and vaccination, comprised 14 items. It included subscales addressing COVID-19 vulnerability, vaccine information and trust, vaccine awareness, and vaccine uptake for children. Specific items in these subscales evaluated concerns about contracting COVID-19, the reliability and safety of vaccine information, awareness of vaccination guidelines for children, and the necessity of vaccines for younger populations. Response options for these items ranged from 0 to 5, with 0 indicating “I don't know” and 5 indicating “Extremely Likely,” and for items C11 and C13, the response options were “Yes” and “No,” with each correct answer scored as 1. This resulted in a maximum possible score of 62.

The Immunization Perception Scale, which focused on general immunization attitudes, consisted of five items initially. This scale included subscales on general vaccine attitudes for children and vaccine hesitancy. Items in these subscales assessed beliefs about vaccine efficacy, parental compliance with vaccination schedules, and instances of vaccine refusal or hesitancy, including refusal of free vaccines provided by the Ministry of Health. For the Immunization questionnaire, the response options were “Yes” and “No,” with each correct answer scored as 1, leading to a maximum score of 5.

The COVID-19 Perception Scale in this study reflects key constructs of the HBM. (Refer to Supplementary Table 3 for mapping of subscales onto HBM Constructs.) Items in the “COVID-19 Vulnerability” subscale assess perceived susceptibility and severity. The “Vaccine Information and Trust” subscale captures perceived benefits, barriers, and self-efficacy related to vaccine knowledge and acceptance. The “Vaccine Awareness” subscale corresponds to cues to action, measuring respondents' awareness of eligibility and access. Finally, the “Vaccine Uptake for Children” subscale evaluates parents' perceived benefits and self-efficacy in making vaccination decisions for their children. Similarly, the Immunization Perception Scale reflects general attitudes and hesitancy, mapping onto the constructs of perceived benefits, barriers, and self-efficacy pillars of HBM. By anchoring the questionnaire development and interpretation within the HBM, this study provides a structured and theory-driven approach to understanding vaccine-related behaviors in a post-pandemic context.

### 2.4 Data analysis

Data analysis was performed using IBM SPSS Statistics version 26.0 (IBM Corp., Armonk, NY, USA). The data analysis for the study was conducted in several stages to ensure a thorough understanding of the participants' perceptions and attitudes toward COVID-19 vaccination and general immunization, as well as the relationships between demographic variables and these perceptions.

#### 2.4.1 Descriptive statistics

Descriptive statistics were employed to summarize and present the results. Categorical items related to the sociodemographic profile of the participants were reported as counts and percentages. For the COVID-19 Perception Scale and the Immunization Perception Scale, descriptive statistics were calculated for all items. These statistics included the mean scores, standard deviations (SD), and the distribution of responses across each response option. This analysis helped to summarize the central tendencies and dispersions of the scores on each subscale, providing a clear picture of parental perceptions and attitudes toward COVID-19 vaccination and general immunization.

#### 2.4.2 Inferential statistics

Non-parametric tests were employed to assess the relationships between demographic variables and the various subscales of COVID-19 perception and general immunization attitudes. Specifically, the Mann–Whitney *U* test and Kruskal-Wallis test were used due to the potential for non-normal distribution of scores. The Mann–Whitney *U* test was used to compare differences between two independent groups (e.g., gender) on each subscale of COVID-19 perception and general immunization attitudes. The Kruskal–Wallis test was used to compare differences between more than two independent groups (e.g., age groups, educational background) on each subscale.

#### 2.4.3 Correlation analysis

Additionally, Spearman's rank-order correlation was employed. Spearman's correlation was chosen as it is less sensitive to outliers and does not assume normal distribution. This non-parametric method allowed for the assessment of monotonic relationships between the ranks of demographic factors (such as age, economic status, and number of children) and the perception scores. These coefficients provided insights into the direction and strength of associations, helping to highlight how changes in demographic variables are associated with changes in perceptions toward COVID-19 vaccination and general immunization among parents.

#### 2.4.4. Visualization

To visually represent the distribution of responses across various questions within the perception subscales, lateral stacked bar graphs were created. These visualizations facilitated a clearer understanding of the distribution and variation in responses, making it easier to identify patterns and trends in the data.

For all statistical analyses, a significance level of p < 0.05 was adopted as the threshold for determining statistical significance. Any p-values below this threshold were considered statistically significant. This comprehensive data analysis approach ensured a robust understanding of parental perceptions toward COVID-19 vaccination and general immunization, and the factors influencing these perceptions.

### 2.5 Ethical considerations

The study obtained ethical approval for the study was obtained from the respective Institutional Review and Ethics Boards (CMH Ref No: ERC#.30/ERC/CMH/LMC, FRPMC Ref No: FRPMC-IRB-2024-52). The research adhered to ethical standards outlined in the Declaration of Helsinki of the World Medical Association for trials involving humans, as per the authors' statements. The consent form was given to the participants, which contained information about the study's purpose, the voluntary nature of their participation, and the measures taken to protect their privacy and confidentiality. Participants were made aware of their right to withdraw from the study at any point without consequences. Additionally, it contained a debriefing statement, promoting transparency and communication. Data security measures were rigorously implemented to safeguard participant information. To ensure the privacy and confidentiality of participants, all data collected was de-identified and stored securely. Personal information such as names, contact details, or other identifiers was not linked to the collected data. Only the research team had access to the data, and any published results or reports would not contain any information that could identify individual participants. The data was stored on secure servers and password-protected computers.

## 3 Results

The baseline characteristics of the study population are summarized in Table 1. The majority of the respondents were male (64.1%), age distribution revealed that 42.3% were between 20 and 34 years, followed by 35–49 years, and 50–65 years. Regarding the relationship with children, fathers represented 64.8% of the respondents. In terms of educational background, a significant majority had a high school education or above (93.6%). The economic status indicated that the majority had incomes ranging from 50,000 to 150,000. The number of family members varied, with a smaller segment in our cohort (5.4%) having 9 to 12 members. Most families (68.8%) had 1 to 3 children. Half of the respondents (50%) indicated that someone in their family, including themselves, had been diagnosed with COVID-19, with an equal proportion (50%) reporting isolation due to COVID-19. Similarly, 50% indicated that someone in their family had tested positive for COVID-19.

**Table 1:** Baseline characteristics.

**Baseline variables**	**Frequency**	**Percentage (%)**
**Gender**		
Male	191	64.1
Female	107	35.9
**Age (years)**		
20–34 years	126	42.3
35–49 years	91	30.5
50–65 years	81	27.2
**Relationship with child/children**		
Father	193	64.8
Mother	105	35.2
**Educational background**		
Middle school and below	19	6.4
High school and above	279	93.6
**Economic status: household total income**		
< 50,000	57	19.1
50,000–150,000	134	45
>150,000	107	35.9
**The number of family members**		
1–4 members	102	34.2
5–8 members	180	60.4
9–12 members	16	5.4
**No of children**		
1–3 children	205	68.8
4–6 children	87	29.2
**Grandparents cohabitant**		
No	192	64.4
Yes	106	35.6
**Co-morbs in family**		
No	71	23.8
Yes	227	76.2
**CO-morbs in child**		
No	211	70.8
Yes	87	29.2
**Is there anyone in your family, including yourself, diagnosed**		
**with COVID-19?**		
No	149	50
Yes	149	50
**Is there anyone in your family, including yourself, who got**		
**isolated due to COVID-19?**		
No	150	50.3
Yes	148	49.7
**Is there anyone in your family, including yourself, who has**		
**tested positive with COVID-19?**		
No	149	50
Yes	149	50

The descriptive statistics for the measured scales assessing parental perceptions toward COVID-19 vaccination and general immunization post-pandemic are detailed in Tables 2, 3. The COVID-19 Perception Scale revealed that parents had an overall average score of 66.6%, with a mean score of 41.3 and a standard deviation of 10.1. Within this scale, the subscales showed varied perceptions. The COVID-19 Vulnerability subscale indicated that parents scored an average of 67.9%, reflecting a moderate perception of their susceptibility to COVID-19, with a mean score of 13.6 (SD = 4.5). The COVID-19 Vaccine Information and Trust subscale had the highest average score of 71.2%, suggesting a relatively high level of trust and information about the COVID-19 vaccine, with a mean score of 17.8 (SD = 6.0). Conversely, the COVID-19 Vaccine Awareness subscale showed a lower average score of 40.0%, indicating a significant gap in vaccine awareness, with a mean score of 2.4 (SD = 1.2). Furthermore, the COVID-19 Vaccine Uptake for Children subscale demonstrated that parents scored an average of 35.5%, revealing limited willingness to vaccinate their children against COVID-19, with a mean score of 3.9 (SD = 1.6).

**Table 2:** Descriptive statistics of measured scales.

**Scales**	**Percentage (%)**	**Mean**	**Std. deviation**
**COVID-19 perception scale**	66.6	41.3	10.1
Subscale 1: COVID-19 vulnerability	67.9	13.6	4.5
Subscale 2: COVID-19 vaccine information and trust	71.2	17.8	6.0
Subscale 3: COVID-19 vaccine awareness	40.0	2.4	1.2
Subscale 4: COVID-19 vaccine uptake for children	35.5	3.9	1.6
**Immunization perception scale**	66.6	3.3	1.6
Subscale 1: general vaccine attitudes for children	81.0	1.6	0.7
Subscale 2: vaccine hesitancy	57.0	1.7	1.2

**Table 3:** Mean of distribution of responses across measured scales.

**Questions asked across scales**	**Mean**	**Std. deviation**
**COVID-19 perception scale**		
**Subscale 1: COVID-19 vulnerability**		
**C1**: My family or I could get COVID-19	3.54	1.30
**C2**: I'm worried that I or someone in my family might get COVID-19	3.31	1.40
**C3**: There are members in my family who can get a severe course if they get COVID-19	3.39	1.46
**C4**: I think that I and my child(ren) are vulnerable to COVID-19	3.34	1.36
**Subscale 2: COVID-19 vaccine information and trust**		
**C5**: How likely is it that you find yourself searching for information about COVID-19 vaccines actively?	3.51	1.53
**C6**: How likely would you think the information about COVID-19 vaccines is reliable?	3.57	1.27
**C7**: How likely would you think that COVID-19 vaccines are preventive?	3.6	1.35
**C8**: How likely would you think that COVID-19 vaccines are safe?	3.46	1.42
**C9**: How likely would you get vaccinated if a vaccine against COVID-19 were available?	3.64	1.59
**Subscale 3: COVID-19 vaccine awareness**		
**C10**: How likely do you think you are aware of the COVID-19 vaccines?	2.07	0.99
**C11**: Currently, children under the age of 18 in Pakistan are not eligible for the COVID-19 vaccination. Did you know this?	0.33	0.47
**Subscale 4: COVID-19 vaccine uptake for children**		
**C12**: If a vaccine against COVID-19 were available, how likely would you get your children vaccinated?	3.64	1.54
**C13**: How likely would you think that COVID-19 vaccines are needed for children and adolescents under 18 years old?	0.66	0.47
**C14:** If a vaccine against COVID-19 were available for children, how likely do you think older children should be vaccinated first due to their outdoor exposure?	3.21	1.59
**Immunization perception scale**		
**Subscale 1: general vaccine attitudes for children**		
**V1**: Do you believe that vaccines can protect children from serious diseases?	0.85	0.36
**V2**: Do you think that most parents like you have their children vaccinated with all the recommended vaccines?	0.77	0.43
**Subscale 2: vaccine hesitancy**		
**V3**: Have you ever been reluctant or hesitated to get a vaccination for your child?	0.52	0.50
**V4**: Have you ever refused a vaccination for your child?	0.64	0.48
**V5**: Have you ever refused a vaccine offered free of charge by the Ministry of Health for your child?	0.56	0.50

The Immunization Perception Scale showed that parents had an overall average score of 66.6%, with a mean score of 3.3 and a standard deviation of 1.6. Within this scale, the General Vaccine Attitudes for Children subscale exhibited a high average score of 81.0%, indicating generally positive attitudes toward vaccinating children, with a mean score of 1.6 (SD = 0.7). However, the Vaccine Hesitancy subscale had an average score of 57.0%, highlighting a moderate level of hesitancy toward vaccination, with a mean score of 1.7 (SD = 1.2).

**Figure 1:**
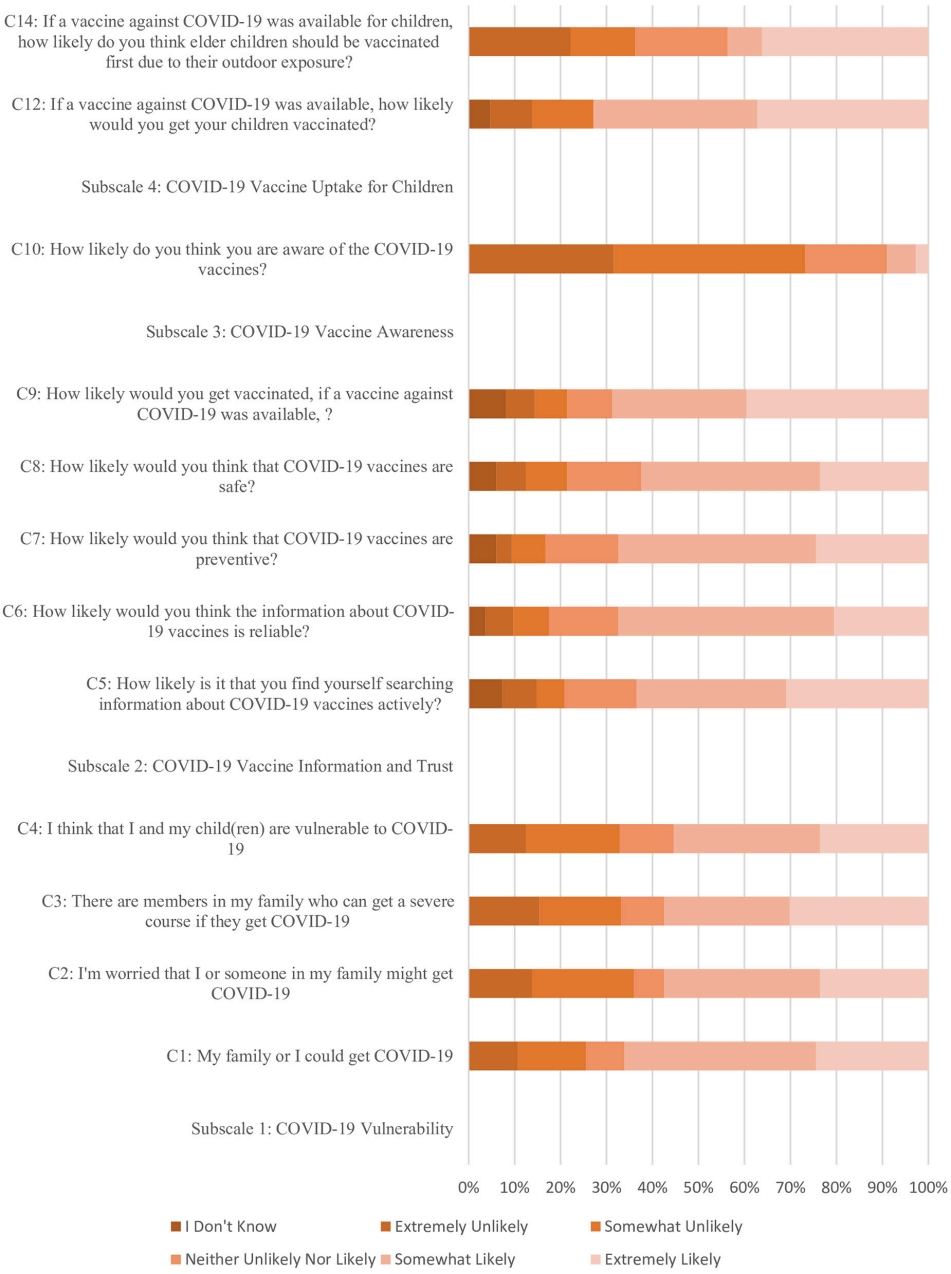
Distribution of responses across COVID-19 perception scale.

**Figure 2:**
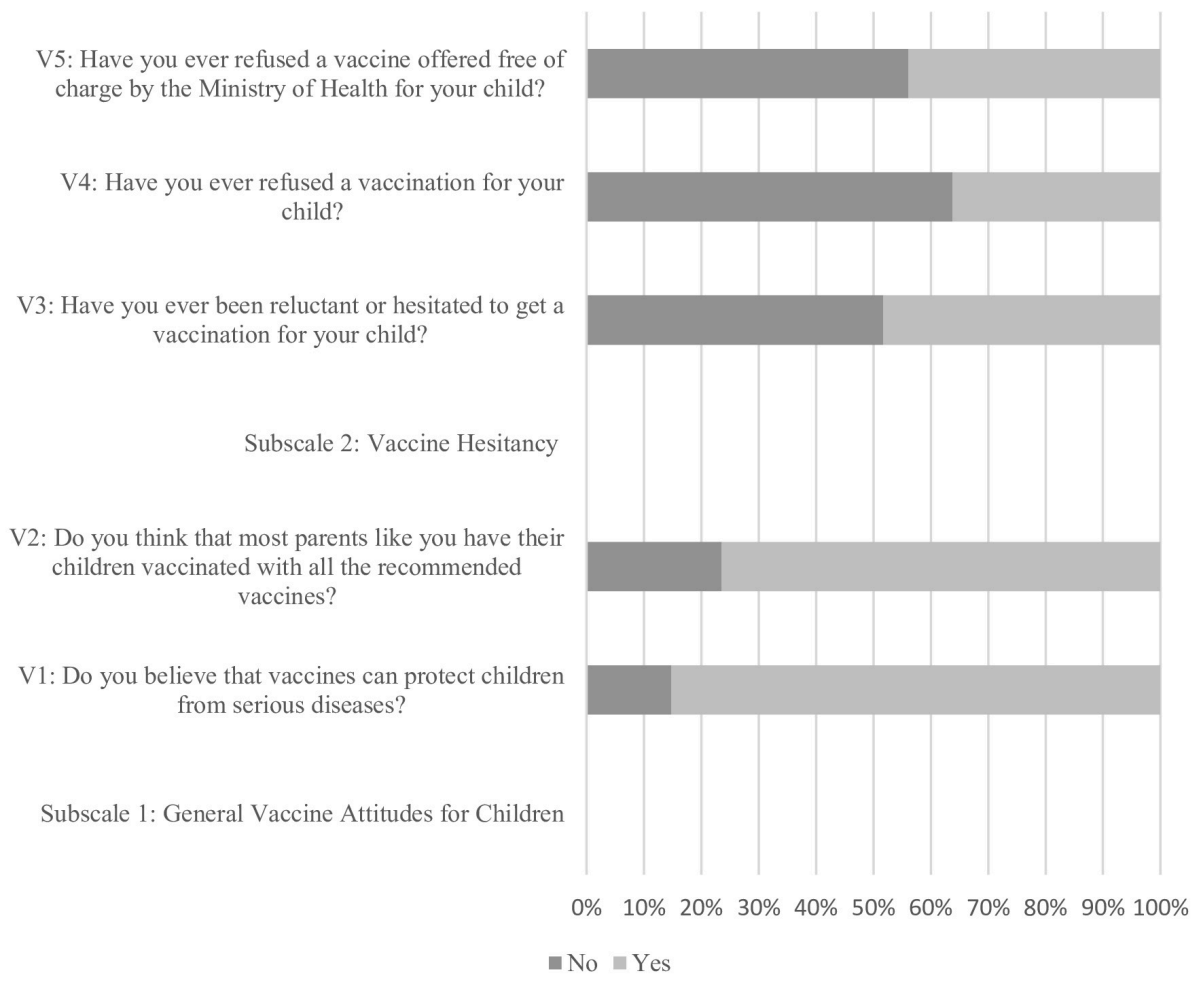
Distribution of responses across immunization perception scale.

The detailed distribution of responses across the measured scales, assessing parental perceptions toward COVID-19 vaccination and general immunization post-pandemic, is presented in Figures 1, 2 (Also presented in tabulated form in Supplementary Tables 4, 5). The COVID-19 Vulnerability subscale indicates that parents generally perceive a moderate level of vulnerability to COVID-19, with items such as “My family or I could get COVID-19” (Mean = 3.54, SD = 1.30) and “I'm worried that I or someone in my family might get COVID-19” (Mean = 3.31, SD = 1.40). Additionally, concerns about severe courses of illness (Mean = 3.39, SD = 1.46) and overall vulnerability of the children (Mean = 3.34, SD = 1.361) were moderately high.

The COVID-19 Vaccine Information and Trust subscale reveals a moderate level of active information searching (Mean = 3.51, SD = 1.53) and trust in the information about COVID-19 vaccines (Mean = 3.57, SD = 1.27). The perception of vaccines being preventive (Mean = 3.6, SD = 1.35) and safe (Mean = 3.46, SD = 1.42) is also moderately high, and the likelihood of getting vaccinated if a vaccine were available scored an average of 3.64 (SD = 1.59). For the COVID-19 Vaccine Awareness subscale, the likelihood of being aware of COVID-19 vaccines is relatively low (Mean = 2.07, SD = 0.99). Awareness about the ineligibility of children under 18 for vaccination in Pakistan scored a low average (Mean = 0.33, SD = 0.47).

The COVID-19 Vaccine Uptake for Children subscale shows a moderate to high likelihood of parents vaccinating their children if a vaccine were available (Mean = 3.64, SD = 1.54). The necessity of vaccines for children and adolescents under 18 years is perceived as moderately important (Mean = 0.66, SD = 0.47), and preference for elder children to be vaccinated first is also moderate (Mean = 3.21, SD = 1.59). The General Vaccine Attitudes for Children subscale indicates a strong belief in the protection vaccines offer against serious diseases (Mean = 0.85, SD = 0.36) and a general agreement that most parents ensure their children receive all recommended vaccines (Mean = 0.77, SD = 0.43). The Vaccine Hesitancy subscale reveals a moderate level of hesitancy (Mean = 0.52, SD = 0.501), with not being reluctant to vaccinate their children scoring higher (Mean = 0.64, SD = 0.482). Non-refusal of free vaccinations offered by the Ministry of Health also showed a moderate level (Mean = 0.56, SD = 0.497).

The association between the measured scales and baseline profiles is presented in Table 4, highlighting significant relationships between certain demographic factors and parental perceptions toward COVID-19 vaccination and general immunization. For the Total COVID-19 Perception Scale, the data indicate that gender, age, relationship with the child/children, educational background, economic status, number of family members, number of children, grandparents cohabiting, and comorbidities in the family do not have statistically significant associations. Significant associations were found with anyone in the family, including the respondent, who had been diagnosed with COVID-19 (*p* < 0.01), had been isolated due to COVID-19 (*p* < 0.01), or had tested positive for COVID-19 (*p* < 0.01).

**Table 4:** Association of measured scales against baseline profile (*p*-value).

**Baseline profile**	**COVID-19 vulnerability**	**COVID-19 vaccine information and trust**	**COVID-19 vaccine uptake and awareness**	**COVID-19 vaccination for children**	**Total COVID-19 perception scale**	**General vaccine attitudes for children**	**Vaccine hesitancy**	**Total immunization perception scale**
Gender	0.622	0.222	0.546	0.881	0.65	0.853	0.158	0.217
Age (years)	0.268	0.028	0.001	0.004	0.19	0.037	0.001	< 0.01
Relationship with Child/Children	0.371	0.377	0.135	0.5	0.799	0.566	0.475	0.43
Educational Background	0.975	0.745	0.005	0.147	0.652	0.781	0.012	0.032
Economic Status: Household Total Income	0.911	0.838	0.001	0.044	0.656	0.961	0.071	0.067
The Number of Family Members	0.495	0.086	0.219	0.855	0.129	0.255	0.118	0.124
No of Children	0.018	0.875	< 0.01	0.508	0.298	0.202	0.044	0.142
Grandparents Cohabitant	0.615	0.998	0.367	0.089	0.361	0.518	0.937	0.964
Co-Morbs in family	0.867	0.433	0.732	0.324	0.727	0.718	0.362	0.579
Co-Morbs in Child	0.368	0.026	< 0.01	0.215	0.077	0.009	0.001	< 0.01
Anyone diagnosed with COVID-19?	< 0.01	0.052	0.203	0.041	< 0.01	0.013	0.175	0.038
Anyone isolated due to COVID-19?	< 0.01	0.122	0.409	0.076	< 0.01	0.016	0.191	0.048
Has anyone tested positive with COVID-19?	< 0.01	0.106	0.94	0.058	< 0.01	0.003	0.029	0.004

For the Immunization Perception Scale, significant associations were found with age (*p* < 0.01), educational background (*p* = 0.032), and the presence of co-morbidities in children (*p* < 0.01). However, gender (*p* = 0.217), relationship with the child/children (*p* = 0.43), economic status (*p* = 0.067), number of family members (*p* = 0.124), number of children (*p* = 0.142), grandparents cohabiting (*p* = 0.964), and co-morbidities in the family (*p* = 0.579) were not significantly associated with the immunization perception scale. Additionally, significant associations were observed with the experience of COVID-19 in the family, including diagnoses (*p* = 0.038), isolation (*p* = 0.048), and positive tests (*p* = 0.004).

Table 5 presents the correlation between measured scales and baseline profile variables, providing insights into the relationships between demographic factors and parental perceptions toward COVID-19 vaccination and general immunization. For the COVID-19 Perception Scale, significant positive correlations were observed with the experience of COVID-19 within the family, including diagnosis (*r* = 0.269, *p* < 0.01), isolation (*r* = 0.264, *p* < 0.01), and positive testing (*r* = 0.252, *p* < 0.01).

**Table 5:** Correlation between measured scales and baseline profile.

**Baseline profile**	**COVID-19 vulnerability**	**COVID-19 vaccine information and trust**	**COVID-19 vaccine uptake and awareness**	**COVID-19 vaccination for children**	**COVID-19 perception scale**	**General vaccine attitudes for children**	**Vaccine hesitancy**	**Immunization perception scale**
Gender	0.029	−0.071	−0.035	−0.009	−0.026	−0.011	−0.082	−0.072
Age (years)	−0.083	−0.137^*^	−0.151^**^	0.193^**^	−0.091	−0.012	0.097	0.083
Relationship with child/children	0.052	−0.051	−0.087	−0.039	−0.015	−0.033	−0.041	−0.046
Educational background	0.002	−0.019	−0.163^**^	0.084	−0.026	−0.016	0.145^*^	0.124^*^
Economic status: household total income	0.005	0.031	−0.220^**^	0.145^*^	0.043	0.013	0.130^*^	0.133^*^
The number of family members	−0.067	−0.112	0.019	0.028	−0.101	0.042	−0.054	−0.017
No of children	−0.139^*^	0.009	0.223^**^	−0.039	−0.061	0.075	−0.118^*^	−0.086
Grandparents cohabitant	−0.029	0	−0.052	−0.099	−0.053	−0.038	0.005	0.003
Co-Morbs in family	−0.01	−0.045	0.02	−0.057	−0.02	0.021	−0.053	−0.032
Co-Morbs in child	−0.052	−0.130^*^	0.248^**^	−0.072	−0.102	−0.152^**^	−0.193^**^	−0.221^**^
Anyone diagnosed with COVID-19?	0.315^**^	0.113	0.074	0.118^*^	0.269^**^	0.144^*^	0.079	0.121^*^
Anyone isolated due to COVID-19?	0.298^**^	0.09	0.048	0.103	0.264^**^	0.140^*^	0.076	0.114^*^
Has anyone tested positive with COVID-19?	0.302^**^	0.094	−0.004	0.11	0.252^**^	0.171^**^	0.127^*^	0.166^**^

^**^Correlation is significant at the 0.01 level (2-tailed).

^*^Correlation is significant at the 0.05 level (2-tailed).

For the Immunization Perception Scale, significant positive correlations were found with educational background (*r* = 0.124, *p* < 0.05), and economic status (*r* = 0.133, *p* < 0.05). A significant negative correlation was observed with co-morbidities in children (*r* = −0.221, *p* < 0.01). Furthermore, significant positive correlations were observed with the experience of COVID-19 within the family, including diagnosis (*r* = 0.121, *p* < 0.05), isolation (*r* = 0.114, *p* < 0.05), and positive testing (*r* = 0.166, *p* < 0.01).

Table 6 shows the results of multivariable regression that family exposure to COVID-19 was the strongest predictor of parental perceptions, with having a family member diagnosed (β = 0.240, *p* < 0.001), isolated (β = 0.210, *p* = 0.002), or testing positive (β = 0.190, *p* = 0.004) significantly increasing COVID-19 perception scores, while demographic factors such as gender, age, education, economic status, and child comorbidities were not significant. In contrast, perceptions toward general immunization were shaped more by socioeconomic and health-related factors: higher education (β = 0.180, *p* = 0.013) and better economic status (β = 0.160, *p* = 0.018) were associated with more positive immunization perceptions, whereas having a child with comorbidities was associated with lower perception scores (β = −0.210, *p* = 0.002). Family COVID-19 experience also contributed positively to general immunization views, with both diagnosis (β = 0.140, *p* = 0.032) and positive test history (β = 0.120, *p* = 0.047) showing significant effects. These findings indicate that lived COVID-19 experience predominantly shapes COVID-specific attitudes, whereas education, socioeconomic resources, and child health status play a larger role in shaping general immunization perceptions.

**Table 6:** Multivariable linear regression predicting perception scores.

**Predictor**	**Model A: COVID-19 perception (β)**	***p*-value**	**Model B: immunization perception (β)**	***p*-value**
Gender	−0.030	0.580	−0.050	0.410
Age	−0.070	0.220	0.030	0.630
Education	0.050	0.390	0.180	0.013^*^
Economic status	0.040	0.440	0.160	0.018^*^
Child comorbidities	−0.080	0.110	−0.210	0.002^**^
Family COVID-19 diagnosis	0.240	< 0.001^**^	0.140	0.032^*^
Family COVID Isolation	0.210	0.002^**^	0.110	0.056
Family positive test	0.190	0.004^**^	0.120	0.047^*^

^*^β = standardized coefficients; ^*^p < 0.05; ^**^p < 0.01.

## 4 Discussion

The findings of this study offer critical insights into post-pandemic parental perceptions of COVID-19 vaccination and general immunization practices within Pakistan—one of the few remaining polio-endemic countries. This context presents a particularly valuable framework for examining the enduring challenges associated with vaccine acceptance and hesitancy. The historical struggle against vaccine-preventable diseases, compounded by sociopolitical, religious, and infrastructural barriers, renders Pakistan a uniquely complex setting in which to study immunization behaviors.

Our study demonstrates a moderate level of positive perception toward COVID-19 vaccination, with an overall score of 66.6%. This is consistent with global literature reporting variable levels of vaccine acceptance depending on contextual factors. For instance, a study reported a 61.6% non-acceptance rate for COVID-19 vaccines, primarily driven by concerns over vaccine safety, distrust in governmental institutions, and fear of adverse effects (15). Conversely, longitudinal research by Sepucha et al. (16) revealed a decline in perceived risk over time following the widespread rollout of vaccines, indicating a public shift toward normalization and vaccine acceptance as more reliable information became available.

Framed through the lens of the HBM, our results align with the theoretical proposition that perceived susceptibility and perceived severity are primary motivators of health-related decision-making. In our study, parents reported moderate concern about contracting COVID-19 and its potential consequences, reflecting a cognitive acknowledgment of risk. This suggests that while parents recognized COVID-19 as a credible health threat, this perception may not have been sufficiently intense or personalized to overcome existing barriers to child vaccination—especially given the lower scores on vaccine uptake subscales. The divergence in perception across different studies may be attributed to contextual heterogeneity, including socio-political trust, religious influences, and the reach of public health messaging. Evidence indicates that regions with higher institutional trust generally report greater vaccine acceptance, whereas environments characterized by misinformation, marginalization, or past programmatic failures tend to exhibit resistance. Sherchan et al. (2024), for instance, documented that perceived healthcare inequities among racial minorities in the United States significantly influenced vaccine hesitancy, underscoring the necessity for inclusive and trust-centered health communication strategies (17).

The public health implications of these findings are substantial. The moderate overall perception score suggests that while many parents demonstrate a basic understanding and acceptance of COVID-19 vaccination, a sizeable subset continues to harbor doubts, misinformation, or uncertainty. According to the HBM, this population may be influenced by perceived barriers (e.g., misinformation, fear of side effects, logistical constraints) and a lack of cues to action, which were not strongly represented in the vaccine awareness or uptake domains of our survey. To address these gaps, targeted health communication interventions that are culturally contextualized, community-driven, and theoretically informed are urgently needed. Such interventions must explicitly address the psychological and structural barriers identified in this and related studies. Moreover, trust-building must be seen not as a byproduct but as a central objective of vaccination campaigns, particularly in populations historically underserved or disillusioned by health systems.

Our findings indicate that the COVID-19 Vaccine Information and Trust subscale yielded the highest average score, reflecting a relatively strong perception of vaccine benefits and high levels of trust in available health information. Within the framework of the HBM, these results align with the construct of perceived benefits, which, alongside perceived susceptibility and severity, significantly influence individuals' likelihood of engaging in preventive health behaviors such as vaccination.

This high trust level is consistent with recent global evidence emphasizing the pivotal role of transparent and reliable health communication in promoting vaccine acceptance. Alzahrani (18) demonstrated that trust in digital health information and public health authorities was a key predictor of vaccine uptake intention, reinforcing the role of perceived credibility in shaping health behaviors. Similarly, D'souza et al. (19) highlighted how historical marginalization and systemic trust deficits negatively impacted vaccine perceptions among underserved communities, underscoring the need to rebuild institutional trust to enhance vaccination rates.

While these findings affirm the effectiveness of current communication strategies, they also reveal disparities in impact across different sociopolitical contexts. For example, Manandhar et al. (20) reported persistently low vaccine confidence in resource-limited settings, attributed to distrust in government-led initiatives and inconsistent messaging. This contrast highlights the critical importance of tailoring trust-building efforts to local contexts, particularly in settings with fragile health systems or prior histories of public health failures. Further supporting this perspective, Bray et al. (21) emphasized that community-university partnerships and grassroots engagement were instrumental in restoring vaccine confidence, particularly in historically hesitant populations. Together, these studies reinforce the notion that generalized messaging is insufficient; rather, localized, culturally sensitive, and partnership-based communication is essential to cultivate enduring trust.

In the context of Pakistan, where misinformation and historical skepticism toward immunization campaigns persist, our findings suggest that building and sustaining trust through transparent communication, leveraging credible local voices, and reinforcing perceived vaccine benefits should remain central components of public health policy.

Our study reveals a notable degree of hesitancy among parents in Pakistan regarding the COVID-19 vaccination of children, with the COVID-19 Vaccine Uptake for Children subscale reflecting a low average score. Despite relatively favorable attitudes toward vaccination in general, this finding highlights a substantial gap when it comes to pediatric COVID-19 vaccine acceptance. Within the framework of the HBM, this low uptake likely reflects strong perceived barriers—a critical construct that can inhibit the translation of health knowledge and intention into actual behavior.

Several specific barriers were evident in our data. Most notably, awareness regarding vaccine eligibility for children was particularly low, suggesting informational deficits. Moreover, ongoing safety concerns, particularly regarding younger age groups, emerged as a key factor contributing to parental reluctance. These concerns are not unique to Pakistan but are widely observed in contexts characterized by limited health literacy, systemic mistrust, and prior negative experiences with immunization campaigns.

Our findings align with international literature emphasizing that pediatric vaccine hesitancy is shaped by a complex interplay of sociocultural, demographic, and experiential factors. For example, Cuccaro et al. (22) reported that in Texas, parental intentions to vaccinate children were significantly influenced by insurance status, prior vaccination behavior, and perceived severity of illness. Similarly, Orangzeb et al. (23) observed in Norway that vaccine uptake varied significantly across immigrant populations, influenced by previous health experiences and structural access barriers. In Calgary, Alberta, demographic characteristics and concerns over the accelerated development of COVID-19 vaccines emerged as critical decision-making variables for parents (24). In contrast, Fayad and Frenck (25) documented increasing pediatric vaccine acceptance in the United States, largely attributed to well-structured public health campaigns, rigorous safety monitoring, and transparent genomic surveillance. These initiatives helped reinforce perceived benefits and reduce perceived risks—key levers in increasing vaccine confidence (25).

By comparison, the persistently low pediatric vaccine acceptance in Pakistan underscores the urgent need for context-specific interventions that directly address parental concerns. These interventions must not only enhance knowledge and awareness but also reduce perceived risks through targeted trust-building strategies, particularly among populations with historical skepticism toward government-led immunization efforts. Engaging pediatricians, community health workers, and local influencers as trusted messengers may prove particularly effective in modifying perceived barriers and improving uptake in this high-risk group.

Our study found that the Immunization Perception Scale yielded an average score, indicating a moderate level of awareness and acceptance of routine immunization among parents in Pakistan. This finding aligns with previous research from various regions within the country, which similarly reported partial awareness and incomplete acceptance of childhood immunization programs. Within the framework of the Health Belief Model (HBM), these outcomes may reflect insufficient cues to action—such as a lack of strong, consistent public health messaging, inadequate provider recommendations, or absence of community-level advocacy—which are essential in prompting vaccine-seeking behavior.

Specifically, our findings of low awareness around vaccine eligibility and scheduling highlight potential communication gaps within national immunization efforts. For instance, Ullah et al. (26) reported that in Balochistan, while 59% of parents acknowledged the importance of vaccines, only 39% demonstrated adequate knowledge regarding specific vaccine-preventable diseases. Similarly, Iqbal et al. (27) observed in South Punjab that although over half of parents were aware of immunization protocols, 42.4% exhibited only moderate understanding. These findings underscore the inconsistencies between attitude and actionable knowledge, a critical distinction in health behavior models.

Further compounding these gaps is the variable presence of self-efficacy—another central HBM construct. While our results suggest generally positive attitudes toward vaccination, this did not uniformly translate into proactive immunization behavior, likely due to limited confidence in navigating the healthcare system, logistical barriers, and poor access to culturally competent guidance. This discrepancy indicates that favorable attitudes alone are insufficient; without adequate structural and psychological support, behavior change remains unlikely.

Educational attainment emerged as a key modulator of immunization perceptions in our study, corroborating findings from other regions in Pakistan. Memon et al. (28), for instance, demonstrated that targeted educational interventions and the involvement of female healthcare providers significantly improved vaccine uptake in rural Sindh. Higher levels of parental education are consistently associated with greater knowledge, lower susceptibility to misinformation, and enhanced decision-making capacity regarding child health. Conversely, vaccine hesitancy remains entrenched in settings characterized by lower literacy, entrenched myths, and limited access to evidence-based information. In Khyber Pakhtunkhwa's newly merged districts, Khan et al. (29) reported that more than one-third of parents exhibited severe vaccine hesitancy, influenced by religious misconceptions, distrust in healthcare systems, and pervasive misinformation. Even in urban centers, such as Karachi, logistical and system-level barriers persist. Memon et al. (30) found that although 80% of mothers initiated childhood immunization, coverage remained inadequate in 30.4% of cases, primarily due to policy confusion and limited physician engagement.

Regression studies helped to clarify the direction of associations, as well as to establish that parental COVID-19 experiences were the most significant predictors of the COVID-19 perception, despite controlling demographic variables. This confirms that perceived susceptibility and severity of perceived exposure are enhanced by lived exposure, which is in line with constructs of the HBM. In case of general immunization, education and economic status raised perceptions, whereas child comorbidities lowered confidence- indicating that parents of the medically vulnerable children are more cautious or hesitant. These results mean that special communication approaches need to focus on the interests of parents with low socioeconomic status or children with health issues.

Taken together, these findings underscore that enhancing immunization outcomes in Pakistan necessitates a multifaceted and context-sensitive approach. While improving knowledge remains essential, it is insufficient in isolation. Sustainable impact requires interventions that not only elevate awareness but also strengthen self-efficacy, amplify culturally resonant cues to action, and systematically address logistical and structural barriers. Public health strategies must be tailored to the distinct needs of both urban and rural populations, ensuring equitable access to reliable information and services. Only by bridging the gap between perception and practice can Pakistan achieve broader and more consistent vaccine coverage, particularly among high-risk and underserved communities.

A nuanced understanding of vaccine perceptions in Pakistan must be contextualized within the historic and ongoing struggle against poliomyelitis, a disease that remains endemic in the country. Persistent challenges—ranging from religious misconceptions and socio-political unrest to deep-seated mistrust of healthcare institutions—have long undermined vaccination efforts. These barriers have cultivated a climate of skepticism toward immunization campaigns, particularly those perceived as externally driven or insufficiently aligned with local values. Consequently, hesitancy toward newer vaccines, including those for COVID-19, is often shaped by this legacy of mistrust and resistance.

Moreover, the rapid expansion of internet access and mobile connectivity has rendered digital platforms a double-edged sword in the dissemination of health information. While these technologies hold transformative potential for public health education, they also serve as fertile ground for misinformation and conspiracy theories, particularly in socio-politically fragmented environments. Combatting this digital infodemic requires strategic engagement of credible community-based actors—including religious scholars, tribal elders, educators, and local influencers—who possess cultural legitimacy and can effectively counter misinformation with scientifically accurate yet socially acceptable narratives.

Our study highlights the multidimensional nature of parental perceptions toward COVID-19 vaccination and routine immunization in Pakistan. Effectively addressing these perceptions calls for an integrated national strategy grounded in community engagement, educational empowerment, and communication transparency. This strategy should leverage trusted social networks, strengthen primary healthcare infrastructure, and deliver consistent, contextually appropriate messaging. Furthermore, investments in health literacy, digital health governance, and public trust-building are critical to shift perceptions and catalyze behavioral change.

Ultimately, overcoming vaccine hesitancy in Pakistan requires more than isolated awareness campaigns; it demands a holistic, long-term public health commitment that confronts historical distrust, systemic inequities, and misinformation ecosystems. By embracing this comprehensive approach, Pakistan can not only enhance immunization uptake but also make meaningful strides toward the eradication of vaccine-preventable diseases such as polio, laying the foundation for a healthier and more resilient future for all children.

## 5 Limitations

This study has several limitations that should be acknowledged. The use of a convenience sampling method from tertiary care hospitals may restrict the generalizability of the findings. Although efforts were made to include participants from diverse demographic backgrounds, but the participants recruited from such settings are often more health-aware, have better healthcare access, and may possess higher educational or socioeconomic status compared to the general population. Consequently, their perceptions and attitudes toward vaccination—both COVID-19 and routine immunization—might differ from those of parents in rural or underserved communities who have limited healthcare exposure or face greater barriers to vaccine access. Additionally, the cross-sectional design captures perceptions at a single point in time, which may not reflect changes in attitudes or behaviors over time. Self-reported data can also introduce response biases, despite measures taken to ensure anonymity and encourage honesty. Moreover, conducting the study in a military hospital setting might have influenced the responses due to the unique characteristics of the population visiting such a facility.

Lastly, while the HBM was used to interpret the study findings, it is important to acknowledge that the questionnaire was not originally developed based on HBM constructs. The application of HBM was conducted *post hoc*, which may limit the completeness or accuracy of construct mapping. As a result, some key theoretical components may not have been fully captured in the instrument. Future studies should consider designing tools explicitly aligned with behavioral models to ensure more robust theoretical integration and analysis.

## 6 Strengths

Despite these limitations, this multi-centered study has several strengths. The rigorous methodology, including pilot testing and validation of the questionnaires, ensured the reliability and validity of the data collected. The inclusion of participants from diverse socioeconomic and educational backgrounds enhances the comprehensiveness of the findings. The high response rate and robust sample size further strengthen the reliability of the results. The sample size and high response rate also strengthen the study's internal validity. Furthermore, by applying the HBM to interpret parental behavior, the study offers a structured, theory-driven lens to understand complex vaccine decision-making processes in a culturally specific context. Lastly, focusing on a polio-endemic country like Pakistan provides valuable insights into vaccine perceptions in a unique context, contributing to the global understanding of vaccine acceptance and hesitancy.

## 7 Future recommendations

Future research should consider longitudinal studies to track changes in parental perceptions over time and understand how different factors influence these perceptions dynamically. Expanding the study to include a more representative sample from various regions of Pakistan would improve the generalizability of the findings. Qualitative research methods, such as focus group discussions and in-depth interviews, could provide deeper insights into the underlying reasons for vaccine hesitancy and acceptance. Furthermore, investigating the impact of targeted educational interventions and trust-building measures on improving vaccine uptake can inform more effective public health strategies. Collaboration with community leaders and leveraging social media positively could help counteract misinformation and build a more informed and trusting public.

## 8 Conclusion

In conclusion, this study reveals a moderate level of positive perception toward COVID-19 vaccination, with significant trust in vaccine information but limited willingness to vaccinate children. General immunization perceptions also showed moderate awareness and acceptance, highlighting gaps in knowledge and ongoing vaccine hesitancy. Framing these findings through the Health Belief Model revealed that while perceived benefits and severity were acknowledged, perceived barriers and a lack of cues to action continue to hinder vaccine uptake. Future research should use longitudinal designs, expand to more diverse populations, and include qualitative methods to better understand vaccine hesitancy. Targeted educational interventions and community engagement are recommended to improve vaccine uptake and public health outcomes in Pakistan.

## Data Availability

The raw data supporting the conclusions of this article will be made available by the authors, without undue reservation.
