# Supplementary material for: Post-pandemic assessment of parental perceptions toward COVID-19 vaccination and general immunization—an insight from polio endemic country
Source: Front Public Health. 2025 Dec 29;13:1627965. doi: 10.3389/fpubh.2025.1627965 (PMC12794569; doi:10.3389/fpubh.2025.1627965)
Supplement: Supplementary file 1 [file Table_1.docx]

**Supplementary Table 1**

| **Supplementary Table 1: Factor Loading of the Asked Questions** | | | | |
| --- | --- | --- | --- | --- |
| **Question** | **Component 1** | **Component 2** | **Component 3** | **Component 4** |
| ***I think COVID-19 is a serious disease*** | **0.328** | **0.367** |  |  |
| *My family or I could get COVID-19* |  | 0.77 |  |  |
| *I'm worried that I or someone in my family might get COVID-19* |  | 0.894 |  |  |
| *There are members in my family who can get a severe course if they get COVID-19* |  | 0.818 |  |  |
| *I think that I and my child(ren) are vulnerable to COVID-19* |  | 0.846 |  |  |
| *Do you search information about COVID-19 vaccines actively?* | 0.788 |  |  |  |
| *How likely would you think the information about COVID-19 vaccines are reliable?* | 0.964 |  |  |  |
| *How likely would you think that COVID-19 vaccines are preventive?* | 0.956 |  |  |  |
| *How likely would you think that COVID-19 vaccines are safe?* | 0.865 |  |  |  |
| *If a vaccine against COVID-19 was available, how likely would you get vaccinated?* | 0.675 |  |  |  |
| *If a vaccine against COVID-19 was available, how likely would you get your children vaccinated?* |  |  |  | 0.588 |
| *How likely do you think you are aware of the COVID-19 vaccines?* |  |  | 0.769 |  |
| *Currently, children under the age of 18 in Pakistan are not eligible for the COVID-19 vaccination. Did you know this?* |  |  | 0.756 |  |
| *Do you think that COVID-19 vaccines are needed for children and adolescent under 18 years old?* |  |  |  | 0.699 |
| *If a vaccine against COVID-19 was available for children, how likely do you think elder children should be vaccinated first due to their outdoor exposure?* |  |  |  | 0.879 |
| Extraction Method: Principal Component Analysis. |  |  |  |  |
| Rotation Method: Promax with Kaiser Normalization. |  |  |  |  |
