# Supplementary material for: Post-pandemic assessment of parental perceptions toward COVID-19 vaccination and general immunization—an insight from polio endemic country
Source: Front Public Health. 2025 Dec 29;13:1627965. doi: 10.3389/fpubh.2025.1627965 (PMC12794569; doi:10.3389/fpubh.2025.1627965)
Supplement: Supplementary file 2 [file Table_2.docx]

**Supplementary Table 2:**

| **Supplementary Table 2: Questionnaire After EFA** |
| --- |
| **COVID-19 Perception Scale** |
| **Subscale 1: COVID-19 Vulnerability** |
| ***C1****: My family or I could get COVID-19* |
| ***C2****: I'm worried that I or someone in my family might get COVID-19* |
| ***C3****: There are members in my family who can get a severe course if they get COVID-19* |
| ***C4****: I think that I and my child(ren) are vulnerable to COVID-19* |
| **Subscale 2: COVID-19 Vaccine Information and Trust** |
| ***C5****: How likely is it that you find yourself searching information about COVID-19 vaccines actively?* |
| ***C6****: How likely would you think the information about COVID-19 vaccines is reliable?* |
| ***C7****: How likely would you think that COVID-19 vaccines are preventive?* |
| ***C8****: How likely would you think that COVID-19 vaccines are safe?* |
| ***C9****: How likely would you get vaccinated, if a vaccine against COVID-19 was available, ?* |
| **Subscale 3: COVID-19 Vaccine Awareness** |
| ***C10****: How likely do you think you are aware of the COVID-19 vaccines?* |
| ***C11****: Currently, children under the age of 18 in Pakistan are not eligible for the COVID-19 vaccination. Did you know this?* |
| **Subscale 4: COVID-19 Vaccine Uptake for Children** |
| ***C12****:* *If a vaccine against COVID-19 was available, how likely would you get your children vaccinated?* |
| ***C13****: How likely would you think that COVID-19 vaccines are needed for children and adolescents under 18 years old?* |
| ***C14:*** *If a vaccine against COVID-19 was available for children, how likely do you think elder children should be vaccinated first due to their outdoor exposure?* |
| **Immunization Perception Scale** |
| **Subscale 1: General Vaccine Attitudes for Children** |
| ***V1****: Do you believe that vaccines can protect children from serious diseases?* |
| ***V2****: Do you think that most parents like you have their children vaccinated with all the recommended vaccines?* |
| **Subscale 2: Vaccine Hesitancy** |
| ***V3****:* *Have you ever been reluctant or hesitated to get a vaccination for your child?* |
| ***V4****: Have you ever refused a vaccination for your child?* |
| ***V5****:* *Have you ever refused a vaccine offered free of charge by the Ministry of Health for your child?* |
