# Supplementary material for: Post-pandemic assessment of parental perceptions toward COVID-19 vaccination and general immunization—an insight from polio endemic country
Source: Front Public Health. 2025 Dec 29;13:1627965. doi: 10.3389/fpubh.2025.1627965 (PMC12794569; doi:10.3389/fpubh.2025.1627965)
Supplement: Supplementary file 3 [file Table_3.docx]

| **Supplementary Table 3: Mapping of Subscales Onto HBM Constructs** | | |
| --- | --- | --- |
| **Subscale** | **Mapped HBM Construct** | **Justification** |
| **COVID-19 Perception Scale:** |  |  |
| *Subscale 1: Vulnerability (C1–C4)* | Perceived Susceptibility + Severity | Measures how at-risk or concerned parents feel about COVID-19 |
| *Subscale 2: Information & Trust (C5–C9)* | Perceived Benefits + Barriers + Self-efficacy | Assesses how much they trust information, perceive vaccine safety/efficacy |
| *Subscale 3: Awareness (C10–C11)* | Cues to Action | Awareness of eligibility and vaccination guidelines can act as behavioral cues |
| *Subscale 4: Uptake for Children (C12–C14)* | Perceived Benefits + Self-efficacy | Likelihood of vaccinating their children, belief in the need for vaccines |
| **Immunization Perception Scale:** |  |  |
| *General Vaccine Attitudes (V1–V2)* | Perceived Benefits | Belief in protection from diseases through vaccines |
| *Vaccine Hesitancy (V3–V5)* | Perceived Barriers + Self-efficacy | Past behaviour reflects psychological barriers or doubts about the ability to decide |
