# Supplementary material for: Post-pandemic assessment of parental perceptions toward COVID-19 vaccination and general immunization—an insight from polio endemic country
Source: Front Public Health. 2025 Dec 29;13:1627965. doi: 10.3389/fpubh.2025.1627965 (PMC12794569; doi:10.3389/fpubh.2025.1627965)
Supplement: Supplementary file 4 [file Table_4.docx]

**Supplementary Table 4**

| **Supplementary Table 4: Distribution of Responses Across Covid-19 Perception Scale** | | | | | | |
| --- | --- | --- | --- | --- | --- | --- |
| **COVID-19 Perception Scale** | **I Don't Know** | **Extremely Unlikely** | **Somewhat Unlikely** | **Neither Unlikely Nor Likely** | **Somewhat Likely** | **Extremely Likely** |
| **Subscale 1: COVID-19 Vulnerability** |  |  |  |  |  |  |
| ***C1****: My family or I could get COVID-19* |  | 32 | 44 | 25 | 124 | 73 |
| ***C2****: I'm worried that I or someone in my family might get COVID-19* |  | 41 | 66 | 20 | 101 | 70 |
| ***C3****: There are members in my family who can get a severe course if they get COVID-19* |  | 46 | 53 | 28 | 81 | 90 |
| ***C4****: I think that I and my child(ren) are vulnerable to COVID-19* |  | 37 | 61 | 35 | 95 | 70 |
| **Subscale 2: COVID-19 Vaccine Information and Trust** |  |  |  |  |  |  |
| ***C5****: How likely is it that you find yourself searching information about COVID-19 vaccines actively?* | 22 | 22 | 18 | 47 | 97 | 92 |
| ***C6****: How likely would you think the information about COVID-19 vaccines is reliable?* | 11 | 18 | 23 | 45 | 140 | 61 |
| ***C7****: How likely would you think that COVID-19 vaccines are preventive?* | 18 | 10 | 22 | 47 | 128 | 73 |
| ***C8****: How likely would you think that COVID-19 vaccines are safe?* | 18 | 19 | 27 | 48 | 116 | 70 |
| ***C9****: How likely would you get vaccinated, if a vaccine against COVID-19 was available, ?* | 24 | 19 | 21 | 29 | 87 | 118 |
| **Subscale 3: COVID-19 Vaccine Awareness** |  |  |  |  |  |  |
| ***C10:*** *How likely do you think you are aware of the COVID-19 vaccines?* |  | 94 | 124 | 53 | 19 | 8 |
| **Subscale 4: COVID-19 Vaccine Uptake for Children** |  |  |  |  |  |  |
| ***C12****: If a vaccine against COVID-19 was available, how likely would you get your children vaccinated?* | 14 | 27 | 40 |  | 106 | 111 |
| ***C14****: If a vaccine against COVID-19 was available for children, how likely do you think elder children should be vaccinated first due to their outdoor exposure?* |  | 66 | 42 | 60 | 22 | 108 |
