# Supplementary material for: Post-pandemic assessment of parental perceptions toward COVID-19 vaccination and general immunization—an insight from polio endemic country
Source: Front Public Health. 2025 Dec 29;13:1627965. doi: 10.3389/fpubh.2025.1627965 (PMC12794569; doi:10.3389/fpubh.2025.1627965)
Supplement: Supplementary file 5 [file Table_5.docx]

**Supplementary Table 5**

| **Supplementary Table 5: Distribution of Responses Across Immunization Scale** | | |
| --- | --- | --- |
| **Immunization Perception Scale** | **No** | **Yes** |
| **Subscale 1: General Vaccine Attitudes for Children** |  |  |
| ***V1****: Do you believe that vaccines can protect children from serious diseases?* | 44 | 254 |
| ***V2****: Do you think that most parents like you have their children vaccinated with all the recommended vaccines?* | 70 | 228 |
| **Subscale 2: Vaccine Hesitancy** |  |  |
| **V3**: *Have you ever been reluctant or hesitated to get a vaccination for your child?* | 154 | 144 |
| **V4**: *Have you ever refused a vaccination for your child?* | 190 | 108 |
| **V5**: *Have you ever refused a vaccine offered free of charge by the Ministry of Health for your child?* | 167 | 131 |
